# Comparative proteomic analysis of different developmental stages of the edible mushroom *Termitomyces heimii*

**DOI:** 10.1186/0717-6287-47-30

**Published:** 2014-07-03

**Authors:** Norasfaliza Rahmad, Jameel R Al-Obaidi, Noraswati Mohd Nor Rashid, Ng Boon Zean, Mohd Hafis Yuswan Mohd Yusoff, Nur Syahidah Shaharuddin, Nor Azreen Mohd Jamil, Norihan Mohd Saleh

**Affiliations:** Agro-biotechnology Institute Malaysia (ABI), c/o MARDI Headquarters, Serdang, Selangor 43400 Malaysia

## Abstract

**Background:**

*Termitomyces heimii* is a basidiomycete fungus that has a symbiotic relationship with termites, and it is an edible mushroom with a unique flavour and texture. *T. heimii* is also one of the most difficult mushrooms to cultivate throughout the world. Little is known about the growth and development of these mushrooms, and the available information is insufficient or poor. The purpose of this study was to provide a base of knowledge regarding the biological processes involved in the development of *T. heimii.* The proteomic method of 2 dimensional difference gel electrophoresis 2D-DIGE was used to determine and examine the protein profiles of each developmental stage (mycelium, primordium and fruiting body). Total proteins were extracted by TCA-acetone precipitation.

**Results:**

A total of 271 protein spots were detected by electrophoresis covering pH 3–10 and 10–250 kDa. Selected protein spots were subjected to mass spectrometric analyses with matrix-assisted laser desorption/ionisation (MALDI TOF/TOF). Nineteen protein spots were identified based on peptide mass fingerprinting by matching peptide fragments to the NCBI non-redundant database using MASCOT software. The 19 protein spots were categorised into four major groups through KEGG pathway analysis, as follows: carbohydrate metabolism, energy metabolism, amino acid metabolism and response to environmental stress.

**Conclusions:**

The results from our study show that there is a clear correlation between the changes in protein expression that occur during different developmental stages. Enzymes related to cell wall synthesis were most highly expressed during fruiting body formation compared to the mycelium and primordial stages. Moreover, enzymes involved in cell wall component degradation were up-regulated in the earlier stages of mushroom development.

## Background

The edible mushroom *Termitomyces heimii* is an agaric-type fungus in the family Lyophyllaceae. It was first discovered in India during the late 1970s [1]. The fungus is also known as “termite mushroom” due to its symbiotic relationship with termites, insects belonging to the subfamily Macrotermitinae. These basidiomycete mushrooms are distributed throughout tropical and subtropical areas of Africa and Asia. Termitomyces fungi are cultivated on a special substrate in the termite nest called the fungus comb [2]. In most *Macrotermes* termites, the fungus comb is composed of their primary faeces, which is mostly undigested dead plant matter [3]. After the termites grow, they feed on the mature parts of the fungus comb and aggregated asexual spores, called fungal nodules. Dead plant materials are thought to be efficiently and strongly degraded in the symbiotic system because fungus-growing termites, unlike many other termites, do not produce faeces rich in organic matter [4]. Thus, the symbiotic fungi are responsible for the efficient decomposition of plant materials by fungus-growing termites. *Termitomyces heimii* is valuable because it has a delicious taste. In addition, the fungus has potential therapeutic uses in blood pressure and blood lipid regulation, the immune response, apoptosis and infection [5]. Its cultivation remains challenging, and numerous worldwide attempts have been made with varying degrees of success. However, all attempts have fallen short as economical cultivation methods [6]. There are limited reports on the biochemical composition of the fungus. This may be due to the difficulty in obtaining wild fruit bodies, as these are seasonal mushrooms. Studies to determine the mechanisms involved in the mushroom’s development are necessary to provide insight into potential fungus cultivation processes. Proteomic approaches, such as two-dimensional difference gel electrophoresis (2D-DIGE) in combination with mass spectrometry (MS), have been widely applied to identify and profile proteins expressed in pathogenic [7, 8] and edible basidiomycetes [9]. A number of studies have convincingly demonstrated that DIGE and MS are complementary proteomics approaches, and the use of both approaches in combination provides deeper insight into the proteome. To better understand the mushroom’s systematic response to environmental stress and protein-protein interactions during different developmental stages, we employed proteomic approaches in this study.

## Results

### 2D-DIGE PAGE at different stages of development

**Figure 1:**
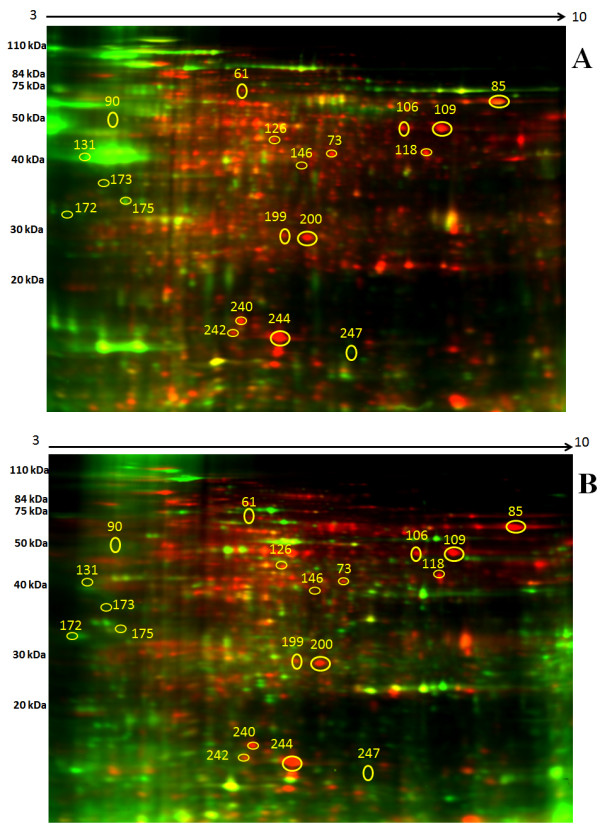
**Representative 2D-DIGE expression maps of the termite mushroom (** ***Termitomyces heimii*** **) labelled with fluorescent dyes (Cy2, Cy3, and Cy5).** The 2D-DIGE image of protein spots compares **(A)** the mycelial proteins labelled with Cy3, the primordial proteins labelled with Cy5, and the internal standard labelled with Cy2. **(B)** The mycelial protein was labelled with Cy3, fruiting body protein was labelled with Cy5, and the internal standard was labelled with Cy2.

To compare the termite mushroom proteome at each of its three developmental stages, total proteins were extracted from the mycelia, primordia and fruiting bodies. For 2D-DIGE, protein extracts were labelled with fluorochromes and mixed prior to separation via 2D electrophoresis. Figures (1A) and (1B) show representative images of the gels from experiments 1 and 2, respectively, in which the separated proteomes can be observed. We normalised and detected the protein spots with Advanced PDQuest 2-D analysis software (Bio-Rad, Hercules, CA, USA). The optical density of each protein spot was evaluated using Student’s t-test. The results from the two experiments reveal approximately 271 protein spots with molecular masses ranging from 10 kDa to 250 kDa and pIs ranging from 3–10. Each stage has a different and unique proteome map (data not shown). For this preliminary study, and to avoid generating a massive amount of data, we selected 19 protein spots with an average ratio value > 1 and *p* value <0.01 that were present in both experiments for identification with MALDI-TOF.

**Figure 2:**
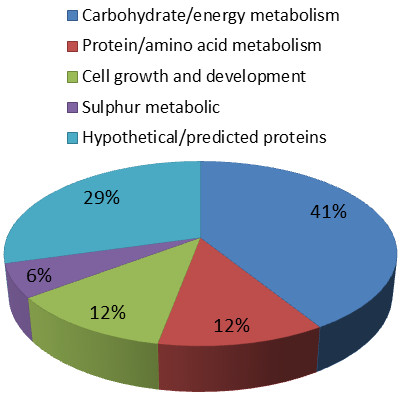
**Classification of proteins according to their biological function.** Assignments were made based on information provided by KEGG pathway analysis.

The 19 proteins were subjected to MS and subsequent database searches; of these, 12 proteins (represented by 14 spots) had a known function, while five proteins had unknown functions (Table 1). Using functional information from the KEGG and Swiss-Prot pathway databases, we assigned biological functions to the identified proteins (Figure 2).

**Table 1:** **Proteins identified from the differential 2D-DIGE analysis obtained from 3 different developmental stages (mycelium, primordium and fruiting bodies) of the edible mushroom** ***Termitomyces heimii***

Spot no	Protein name	Accession no.	^a^Protein MW	Protein p*I*	Peptide count	Protein score	Protein score C.I%	Total ion	Total ion score C.I%	Average ratio value			
										^b^P/M	*p*value	^c^F/M	*p*value
172	Predicted protein [Laccaria bicolor S238N-H82]	gi|170097613	73,362	5.2	8	459	100	437	100	-1.4	0.14	-7.4	0.00057
109	NADP-glutamate dehydrogenase [Laccaria bicolor]	gi|1101027	48,444	6	8	348	100	317	100	5.9	0.026	42.0	0.00042
106	NADP-glutamate dehydrogenase [Laccaria bicolor]	gi|1101027	48,444	6	9	342	100	303	100	1.2	0.58	16.9	0.0021
247	Translation elongation factor 1-alpha [Uromyces polygoni-avicularis]	gi|197726092	17,846	8.6	5	215	100	184	100	5.8	0.0014	-1.7	0.069
199	Cysteine peroxiredoxin [Laccaria bicolor S238N-H82]	gi|170090830	25,089	5.8	5	165	100	95	100	1.7	0.12	8.2	4.50E-06
175	Carbohydrate esterase family 4 protein [Laccaria bicolor S238N-H82]	gi|170093812	27,286	6.2	3	159	100	149	100	10.9	0.00094	3.3	0.0099
200	Cysteine peroxiredoxin [Laccaria bicolor S238N-H82]	gi|170090830	25,089	5.8	5	145	100	104	100	1.8	0.076	33.6	1.20E-06
61	Phosphoglucomutase [Micromonaspusilla CCMP1545]	gi|303273426	64,752	5	6	126	100	114	100	-1.2	0.76	4.1	0.0093
146	Fructose 1,6-bisphosphate aldolase [Laccaria bicolor S238N-H82]	gi|170106499	38,916	5.5	7	126	100	98	100	1.6	0.027	12.0	9.70E-06
118	Predicted protein [Laccaria bicolor S238N-H82]	gi|170100663	51,868	6.6	5	115	100	103	100	17.7	0.003	16.6	0.0016
126	Putative sulfite reductase [Janibacter sp. HTCC2649]	gi|84497034	11,622	6.3	3	97	100	80	100	1.2	0.31	5.4	0.00019
131	Aspartic peptidase A1 [Laccaria bicolor S238N-H82]	gi|170091822	44,125	5	6	86	98	66	99	9.2	0.00012	1.1	0.58
90	Hypothetical protein MPER_03344 [Moniliophthoraperniciosa FA553]	gi|238605335	18,291	8	1	85	97	80	100	19.1	0.00056	1.2	0.59
240	DNA primase [Oceanicolagranulosus HTCC2516]	gi|89070862	69,526	6.8	13	83	96	37	0	47.4	7.00E-07	12.6	2.90E-06
244	(p)ppGppsynthetase I SpoT/RelA [Actinobacillussuccinogenes 130Z]	gi|152978696	70,073	8	7	71	33	55	91	1.2	0.08	47.7	7.20E-07
73	Hypothetical protein MPER_10776 [Moniliophthoraperniciosa FA553]	gi|238582725	28,866	7.2	1	66	0	66	99	65.7	0.00014	37.3	0.00012
242	Metallophosphoesterase [Spirosomalinguale DSM 74]	gi|284036135	26,893	5.8	1	64	-	64	99	-2.4	0.013	5.3	0.0011
85	Hypothetical protein Phum_PHUM233470 [Pediculushumanuscorporis]	gi|242010560	222,895	9.2	14	63	-	63	99	19.9	2.80E-06	99.3	1.60E-06
173	NADH dehydrogenase (quinone) [Opitutus terrae PB90-1]	gi|182412569	45,006	6.8	1	61	-	61	98	12.5	5.80E-05	2.0	3.10E-03

^a^Protein Molecular Weight; ^b^Primordium/Mycelium; ^c^Fruiting bodies/Mycelium; +/-up-regulation/down-regulation.

## Discussion

During the development of all basidiomycete fungi, the vegetative mycelium stage transforms into a fruiting body via an intermediate primordium stage, which is a complicated and critical process [10]. Understanding the molecular mechanisms underlying this process has long been a goal in basidiomycete research. By defining the proteome at each developmental stage, we can obtain a broader understanding of the mushroom fruiting process [11].

This study is one of the first reports of the termite mushroom proteome. Unlike classical proteomic studies that produce large quantities of information, we screened different developmental stages of the termite mushroom for proteins with significantly but consistently altered expression levels during development. Therefore, we focused on 19 spots that passed our screening criteria. NADP-dependent glutamate dehydrogenase (NADP-GDH) is an intermediate enzyme in fungal ammonium absorption and converts 2-oxoglutarate to glutamate [12]. NADP-GDH is positioned at the border of carbon and nitrogen metabolism and is potentially involved in the regulation of these processes [13]. Although the cofactor enzyme NADH dehydrogenase (spot #173) was down-regulated during fruiting body development, we found that NADP-GDH (spot #109 and 106) was slightly up-regulated in the primordia stage compared to the mycelium. However, when we compared its expression between fruiting bodies and primordia, it was similarly, but not significantly, up-regulated. Metallophosphoesterase (spot #242) had a similar expression profile. It was not significantly down-regulated during the primordial stage; however, it was highly up-regulated in fruiting bodies. This was expected, as the levels of cell wall enzymes likely increase after fruiting bodies are harvested in basidiomycetes [14]. Carbohydrate esterase 4 (Spot #175) hydrolyses proteins, especially pectins. We found that it was significantly up-regulated during primordial formation, but its expression significantly decreased during fruiting body formation. It was previously reported that carbohydrate esterase 4 was up-regulated in fungal cells during their interactions with their host cells [15, 16]. In addition, Yang et al. [17] found that carbohydrate esterase was expressed during fruiting body formation of the closely related edible mushroom *Termitomyces albuminosus*, which suggests that termite mushrooms can degrade lignin. The mushroom cooperates with the enzymes produced by the fungus-growing termite to degrade the cellulose and hemicellulose of plant cells, which supports the well-coordinated cooperation hypothesis and potentially explains the symbiotic relationship between the termite and the fungus [18].

We found that translation elongation factor 1-alpha (TEF α1) was up-regulated in the primordial stage compared to the mycelium. While the expression of the protein in the fruiting stage was not significantly down-regulated compared to its expression in the mycelium stage, additional translation factors are likely involved. TEF α1 is thought to play an important and major role in the regulation of cell wall morphogenesis [19]. Similarly, TEF α1 was up-regulated in the basidiomycete *Pleurotus tuberregium* during the transition from mycelium to primordium [20].

Two spots (#199 and #200) represented cysteine peroxiredoxin. Peroxiredoxins are a class of fungal antioxidants that reduce hydroperoxides to alcohols [21]. We found that the protein was highly expressed in the primordia and was significantly higher in fruiting bodies compared to the mycelium. Cysteine peroxiredoxin usually localises to the cytosol and mitochondria. In eukaryotic cells, cysteine peroxiredoxin is highly expressed in various isoforms and, despite being less efficient than catalase, cysteine peroxiredoxin likely contributes to the protection of the plasma membrane against lipid peroxide oxidation [22]. This may be why cysteine peroxiredoxin levels increase during fungal development. Two different proteins involved in the glycolytic pathway, phosphoglucomutase (spot #61) and fructose 1,6 bisphosphate aldolase (spot #146), did not significantly change during the primordia stage; however, both proteins were significantly up-regulated during fruiting body formation. Insignificant changes in the levels of proteins related to the glycolytic pathway may lead to changes in glycolysis, gluconeogenesis, and mitochondrial function, which may be beneficial for nascent fungal cells adapting to an anaerobic environment [23]. Increased levels of these proteins may be necessary for the development of the rigid cell wall during fruiting body formation [24]. Sulphite reductase (spot #126) is another enzyme we detected that slightly changes during primordia formation and significantly changes during fruiting body formation. Although studies have been performed on bacterial cells, there is little information available on the functional role of sulphite reductase in mushroom development, except that it plays a role in sulphur assimilation in yeast during fermentation [25].

**Figure 3:**
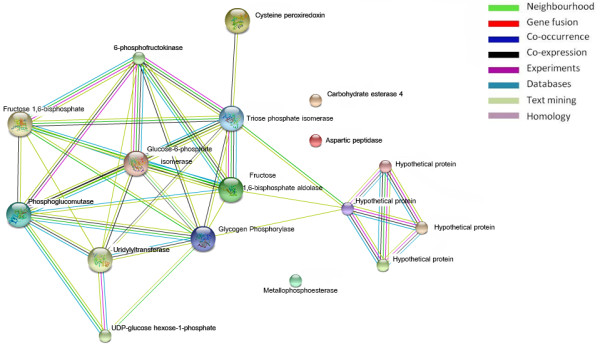
**Simulated functional network of differentially expressed proteins constructed using the STRING database.**

Aspartic peptidase A1 belongs to the protease family. We found that it was highly expressed during primordial development (spot #131), but its expression dramatically decreased during fruiting body formation. The enzyme was previously isolated from the basidiomycete fungus *Piptoporus soloniensis* [26], but it did not have a clear role in mushroom development. It is possible that aspartic peptidase A1 is down-regulated because the termite mushroom is a non-pathogenic fungus. The same expression pattern was observed for DNA primase (spot #240), although it was highly expressed after the mycelium stage, which may be due to the decrease in meiosis of fruiting body cells after a high level of meiosis during the primordial stage [27]. ppGpp guanosine pentaphosphate (spot #244) was also highly expressed during fruiting body development. The enzyme is an alarmone, which is involved in the stringent bacterial response [28], resulting in the inhibition of RNA synthesis when there is a shortage of amino acids. To the best of our knowledge, this is the first report describing the up-regulation of ppGpp guanosine pentaphosphate in basidiomycetes. To further understand the protein-protein interactions that occur during mushroom development, we created a protein network (Figure 3) using the STRING protein interaction database (http://string-db.org/) using the identified proteins with known functions as input with a medium level of confidence [29]. The two proteins involved in the glycolytic pathway, phosphoglucomutase and fructose 1,6-bisphosphate aldolase, had the highest number of interactions (7 and 6, respectively). The interaction network revealed the importance of these two proteins in glycolytic regulation, as these two proteins were connected to other enzymes involved in the pathway, such as glucose-6-phosphate isomerase and triose phosphate isomerase. The proteins with the highest number of connections were those that were up-regulated during fruiting body formation. The expression of these proteins may be necessary to develop the rigid cell wall and rapid cell expansion that are characteristic of fruiting body formation [24]. Cysteine peroxiredoxin was highly expressed during fruiting body formation, and we identified a single connection to triose phosphate isomerase. Triose phosphate isomerase is related to the glycolytic pathway, and this interaction may explain the role of cysteine peroxiredoxin in plasma membrane integrity. Together with cell wall formation, the interaction between triose phosphate isomerase and cysteine peroxiredoxin may be necessary for fruiting body formation [14, 24].

## Conclusion

The identification of proteins with potential roles in *T. heimii* mushroom development could provide useful information regarding fruiting body formation, which is possibly one of the most important developmental events in the life cycle of this mushroom, as it allows the formation of propagative structures. While altered protein expression may be physiologically relevant to the fungus and can facilitate changes in metabolism via different pathways, it may also be directly involved in the fruiting body development of *T. heimii,* a rare, highly desirable, edible seasonal fungus. We are currently working to obtain more information on the proteome of each developmental stage of the fungus by utilising various proteomic tools, such as liquid chromatography-based separation and label-free quantitative proteomics. These tools can be used to ultimately improve the cultivation methods of *T. heimii*.

## Methods

### Samples

**Figure 4:**
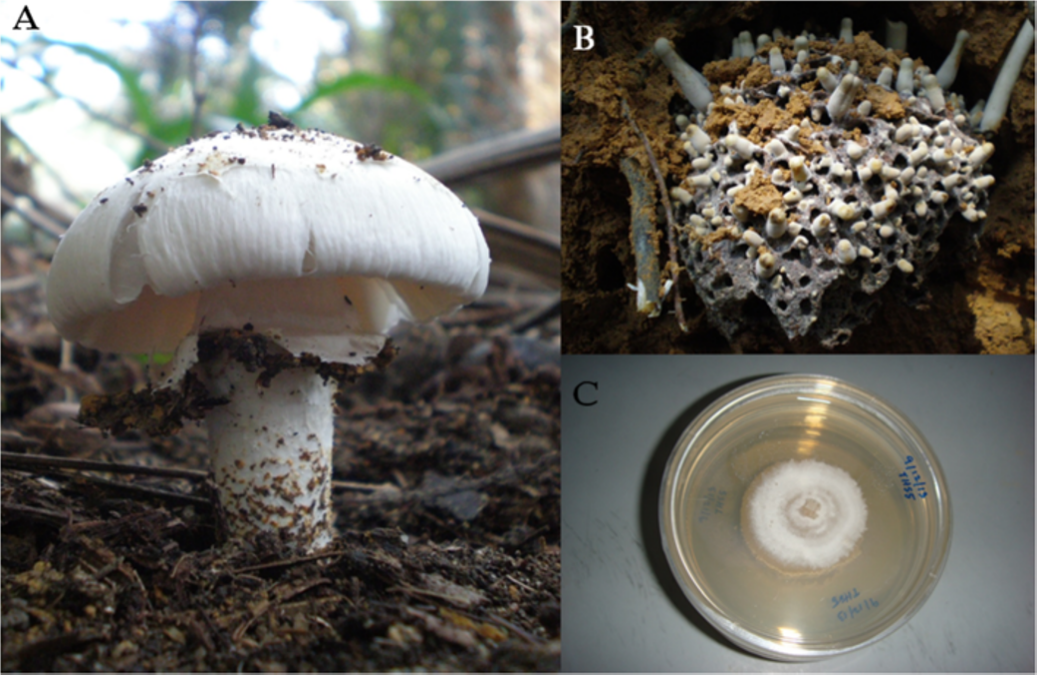
**Termite mushroom during different developmental stages. (A)** Basidiomata of *Termitomyces heimii*. **(B**) Primordia growing on termite nest. **(C)** *Termitomyces heimii* mycelium.

*Termitomyces heimii* were collected during the rainy and wet season at Semenyih (2.9500° N, 101.8500° E), Selangor, Malaysia. Mycelia of *T. heimii* were cultured on modified Hagem Modess (HM) media and incubated for 3 weeks (Figure 4). Samples at different stages of development (mycelia, primordia and fruiting bodies) were immediately frozen by submerging the samples in liquid nitrogen. The samples were then stored at -80°C for further use.

### Protein extraction

Separate samples were ground using a mortar and pestle in liquid nitrogen. Protein extraction was carried out using TCA acetone precipitation [30]. Pellets were resuspended in lysis buffer containing 30 mM Tris–HCl, pH 8.8, 7 M urea, 2 M thiourea and 4% CHAPS. Protein quantitation was conducted with a Bradford kit (Bio-Rad, USA) with bovine serum albumin as a standard.

### 2D-DIGE

#### CyDye labelling

Two experiments with 3 biological replicates each were performed. Each proteome was labelled with an appropriate fluorochrome (Cy2 for the internal standard, Cy3 for the mycelial protein and Cy5 for the primordial protein). In the second experiment, the proteins were labelled similarly to those in the 1st experiment, except that Cy5 was used to label the fruiting body protein as shown in Figure 1B. For each sample, 30 μg protein was mixed with 1.0 μl diluted CyDye, and kept in the dark on ice for 30 min. Samples from each pair were labelled with Cy3 and Cy5, respectively, and an internal standard with Cy2 was run on each gel. The labelling reaction was terminated by adding 1.0 μl 10 mM lysine to each sample and incubating in the dark on ice for an additional 15 min. The labelled samples were then mixed together. The 2× 2-D sample buffer (8 M urea, 4% CHAPS, 20 mg/ml DTT, 2% pharmalytes and a trace amount of bromophenol blue), 100 μl destreak solution and rehydration buffer (7 M urea, 2 M thiourea, 4% CHAPS, 20 mg/ml DTT, 1% pharmalytes and a trace amount of bromophenol blue) were added to the labelling mixture to bring the total volume to 250 μl. The solution was mixed well and spun down before loading the labelled samples into a strip holder.

### IEF and SDS-PAGE

The isoelectric focusing step (IEF) was carried out on a PROTEAN^®^ i12™ IEF cell (BioRad) by applying the samples to 13 cm Immobiline™ DryStrip gels (IPG), with a linear pH range of 3–10 (GE Healthcare). Rehydration was performed at 250 V for 2 hours in the linear mode followed by 250 V constant for 2 hours, 250–5000 V for 4 hours in the linear mode and 5000 V constant for a total of 8 hours and 35 kvh. Upon completion of IEF, the IPG strips were incubated in freshly made equilibration buffer-1 (50 mM Tris–HCl, pH 8.8, 6 M urea, 30% glycerol, 2% SDS, a trace amount of bromophenol blue and 10 mg/ml DTT) for 15 minutes with gentle shaking. The strips were then rinsed in freshly made equilibration buffer-2 (50 mM Tris–HCl, pH 8.8, 6 M urea, 30% glycerol, 2% SDS, a trace amount of bromophenol blue and 45 mg/ml DTT) for 10 minutes with gentle shaking. Next, the IPG strips were rinsed in SDS gel running buffer and placed on the top of the SDS gels (12% gels). The SDS gels were run at 15°C until the dye front ran out of the gels.

### Image scan and data analysis

Gel images were immediately scanned following SDS-PAGE using Typhoon TRIO (GE Healthcare). The scanned images were analysed with PDQuest 2-D analysis software (Bio-Rad), and multiplex gel images were generated by cross-gel analysis using DeCyder software (version 6.5, GE Healthcare). Quantitative and statistical analyses of changes in the spots were performed with DeCyder software (GE Healthcare).

### Protein identification by mass spectrometry

#### Spot picking and trypsin digestion

Spots of interest were picked up using the Ettan Spot Picker (Amersham BioSciences) based on the in-gel analysis and spot picking design by DeCyder software. The gel spots were washed several times and digested in-gel with modified porcine trypsin protease (Trypsin Gold, Promega). The digested tryptic peptides were desalted by Zip-tip C18 (Millipore). Peptides were eluted from the Zip-tip with 0.5 μl matrix solution (α-cyano-4-hydroxycinnamic acid, 5 mg/ml in 50% acetonitrile, 0.1% trifluoroacetic acid, 25 mM ammonium bicarbonate) and spotted on the AB SCIEX MALDI plate (Opti-TOF™ 384 well insert).

### Mass spectrometry

MALDI-TOF MS and TOF/TOF tandem MS/MS were performed on an AB SCIEX TOF/TOF™ 5800 System (AB SCIEX, Framingham, MA). MALDI-TOF mass spectra were acquired in reflectron positive ion mode, averaging 4000 laser shots per spectrum. TOF/TOF tandem MS fragmentation spectra were acquired for each sample, averaging 4000 laser shots per fragmentation spectrum on each of the 7–10 most abundant ions present in each sample (excluding trypsin autolytic peptides and other known background ions).

### Database search

Both the resulting peptide mass spectra and associated fragmentation spectra were submitted to a GPS Explorer workstation equipped with the MASCOT search engine (Matrix science) to search the non-redundant database of the National Center for Biotechnology Information (NCBInr). Searches were performed without constraining protein molecular weight or isoelectric point, with variable carbamidomethylation of cysteine and oxidation of methionine residues and with one allowed missed cleavage site. Candidates with either a protein score C.I.% or ion C.I.% more than 95 were considered significant.

## Abbreviations

2D-DIGE: 2 Dimensional difference gel electrophoresis
CHAPS: 3-[(3-Cholamidopropyl) dimethylammonio]-1-propanesulfonate
TCA: Trichloroacetic acid
MS: Mass spectrometry
MALDI TOF/TOF: Matrix-assisted laser desorption/ionization/ time-of-flight
KEGG: Kyoto encyclopedia of genes and genomes
DTT: Dithiothreitol
IEF: Isoelectrical focusing
IPG: Immobilized pH gradient
STRING: Search Tool for the retrieval of interacting genes.
